# Open Reduction Internal Fixation Poststernotomy Mediastinitis

**DOI:** 10.1155/2013/571685

**Published:** 2013-07-17

**Authors:** Hani Sinno, Tassos Dionisopoulos

**Affiliations:** Division of Plastic Surgery, Department of Surgery, Jewish General Hospital, 3755 Côte-Sainte-Catherine Road, A500, Montreal, QC, Canada H3T 1E2

## Abstract

*Introduction.* Mediastinitis has been reported to complicate 5% of sternotomy surgery. We have adopted an open reduction and rigid internal fixation (ORIF) approach during the conventional rescue surgery in the treatment of mediastinitis. *Methods.* A retrospective review was performed to compare the outcomes of patients that had an ORIF to correct postoperative mediastinitis following median sternotomy. These were compared with the outcome of the patients that did not undergo ORIF. *Results.* In the 5-year study period, we reviewed 35 mediastinitis patient charts. Postoperatively, the ORIF patient group remained in the Intensive Care Unit (ICU) and on a ventilator for a mean of 1.5 and 0.75 days, respectively. Patients treated without ORIF spent significantly more days in the ICU (mean of 7.5 days, *P* < 0.05) and on a ventilator (mean of 2.15 days, *P* = 0.1). Furthermore, it was found that none of the patients (0%) who underwent ORIF complained of any postoperative sternal instability or pain. Preoperatively, however, these rates were as high as 72%. *Conclusions.* In the select patient, ORIF can be a safe option in the management of mediastinitis, which we have shown to significantly decrease morbidity and mortality by providing anatomic reduction as well as physiologic stabilization. We have shown that ORIF will improve the quality of life of the patient by minimizing abnormal sternal mobility and pain and will also decrease inpatient costs by decreasing days spent in the ICU and ventilator dependence.

## 1. Introduction

Since the introduction of the median sternotomy technique for cardiac surgery in the 1950's and its support by surgeons such as Gerbode et al. [1] and Julian et al. [2], it has become the standard approach for open access to the anterior mediastinum. Although the wound complications of such operative techniques are relatively small (i.e., wound infections reach up to 2.1% and bony nonunion up to 5.1% of all cases), their consequences are extremely detrimental. Mortality rates have been reported up to 100% of all cases if untreated [3–9].

Wound infections have ranged from being benign involving the skin edges to more serious abscess formation involving skin, subcutaneous fat, bone, cartilage, and mediastinum structures. In addition, the separation of the bony sternum and manubrium after median sternotomy is referred to as sternal dehiscence. This phenomenon may be associated with infection, sternal wires shearing through the bone, wire rupture, sternal necrosis, sternal nonunion, mechanical stresses, and/or trauma. The transverse sternal fractures created by the sternal wires and sternal necrosis are often associated with sternal wound infection. With respect to the more superficial infections, the fractures can be associated with a sterile sternal dehiscence. Furthermore, it has been observed that infections along with sternal dehiscence appear more acutely with the symptoms being attributed to the infection. On the other spectrum, the sternal dehiscence not associated with infection appears later in the postoperative period with symptoms being primarily with sternal motions pain. In summary the literature utilizes terms such as sternal wound infection, median sternotomy dehiscence, sternal dehiscence, and poststernotomy mediastinitis interchangeably to describe these wound complications.

As mediastinitis poststernotomy complications entail significant morbidity, increased hospital costs, and mortality rates of 50–100%, many management techniques have been described [4]. In 1963 Shumacker and Mandelbaum reported their methods of continuous antibiotic irrigation providing significant mortality rates [10]. In 1976 Lee et al. [11] described the omental flap, and, in 1980, Jurkiewicz et al. [12] described the pectoralis major advancement flaps for soft tissue coverage further decreasing the mortality and morbidity rates. However, even with these methods of management, complications such as necessity of prolonged sedation, prolonged mechanical ventilation, and prolonged ICU stays remained. Furthermore, issues such as the need for medical paralyzation, increased work of breathing, chronic chest or shoulder pain secondary to the bony nonunion were not addressed. To address such issues a new way of management was developed. In 1994 Gottlieb et al. [13] described and reviewed their experience with a new technique of sternal salvage based on osseous quantitative bacteriologic assessment and rigid fixation in 29 patients with postoperative mediastinitis. This group described the use of transverse titanium miniplates or mesh to fixate the bony nonunion to correct the underlying issues. Furthermore, they discovered that a radical sternal debridement may not be necessary in all patients with postoperative mediastinitis following median sternotomy. In addition, they noted that sternal salvage can be safely and reliably achieved with a combination of clinical assessment of vascularity and osseous quantitative bacteriologic assessment using an anatomic reduction of the viable sternal segments even in severely osteoporotic bone.

More recently, Chase et al. [14] described their technique of management of poststernotomy mediastintis in 30 patients. In contrast to Gottlieb's transverse plates, Chase et al. demonstrated their use of longitudinal placed titanium plates on each hemisternum for bony fixation. In summary, their experience reports an alternative, single-stage technique of debridement, internal fixation of the sternum, pectoralis major musculocutaneous advancement flaps, and primary closure used in patients with sternal dehiscence following median sternotomy. Similarly they conclude that a stable, closed median sternotomy wound with minimal morbidity and mortality is accomplished in one procedure that can be used in any type of sternal dehiscence, whether infected or sterile, acute or chronic. 

Mitra et al. [15] presented their 4-year experience using a composite technique for salvage closure of difficult sternotomy wounds. They placed stainless steel wires immediately beneath titanium reconstruction plates affixed to the superficial aspect of each hemisternum. Six patients were reported to undergo this technique and have achieved sternal closure with no complications. Furthermore, just recently, Cicilioni et al. [16] published their experience of their technique of open reduction and rigid internal fixation using titanium plates in 50 patients achieving similar results of 98% bony union.

In light of such advancements, we decided to retrospectively review our results of the past 5 years since we began to utilize this single-stage technique of debridement, internal fixation of the sternum, pectoralis major musculocutaneous advancement flaps, and primary closure used in patients with sternal dehiscence following median sternotomy. Furthermore, for the first time to our knowledge we have addressed the issue of sternal pain and sternal motion with this technique as compared to the management of mediastinitis with no internal fixation.

## 2. Operative Technique

**Figure 1:**
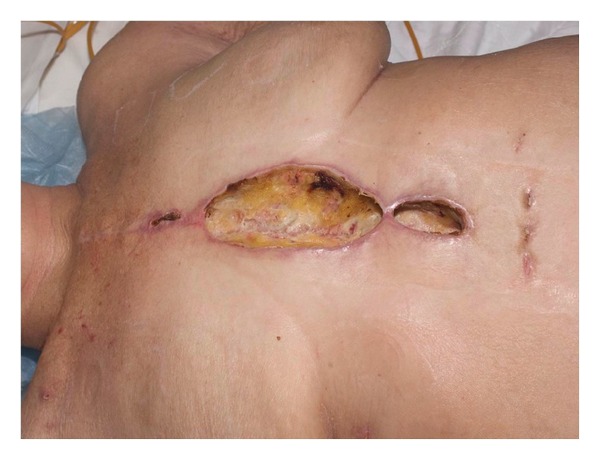
Sternal dehiscence postmedian sternotomy as detected by the cardiothoracic surgeon.

Patients are referred to plastic surgery service by the cardiothoracic surgeon who performed the original sternotomy procedure. Any signs of sternal wound erythema, infection, dehiscence, or sternal “clicking” (Figure 1) are regarded by the cardiac surgeons as possible hints of mediastinitis. Consequently, we have been treating these complications sooner and sooner providing superior outcomes as the infections are caught relatively earlier.

General anesthesia is performed for the patient undergoing the operation. The arms of the patient are not placed on any arm board but are padded and tucked alongside the supine body as to prevent stretching on the pectoralis muscles which would otherwise create a reduction of the sternal separation. Confined to sternal technique, the sternotomy wound is excised including all skin, subcutaneous tissue, any necrotic-appearing tissue, and chronic granulation tissue present down to the level of the sternal bone. By this, the chronic wound would be converted to an acute one. After that, the existing sternal wires are excised, and all infected and nonviable tissue is vigorously debrided. All necrotic and nonviable bones and cartilages are debrided until they are free of devitalized tissue and bleeding. Bone biopsy and wound exudate are sent for definitive culture as low-grade chronic osteomyelitis must be excluded as causal factor in the nonunion. The entire wound is subsequently pulse irrigated with 3 liters of warm normal saline containing 50,000 units of bacitracin through a pressurized pulse-irrigation system. 

**Figure 2:**
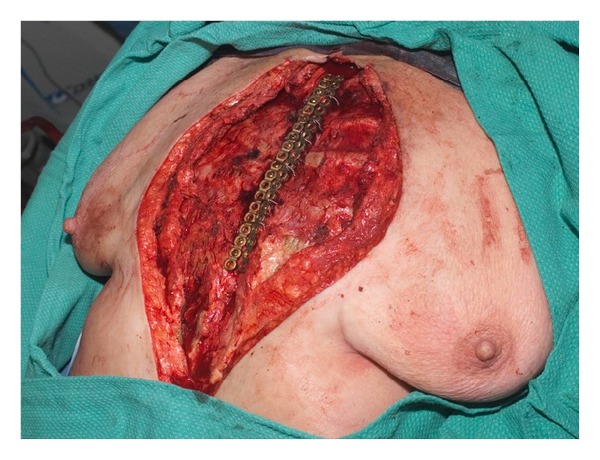
Stainless steel reconstruction plates are manipulated and secured vertically to each remaining hemisternum after sufficient debridement and pulse irrigation. The sternum is reduced with bone-approximating clamps, wires reapproximate, and tightened the plates in an anatomical reduced position.

The two sternal halves are reduced temporarily using Bailey rib approximators. Subsequently, stainless steel mandibular reconstruction plates (2.7 mm) are tailored to lie vertically along each remaining hemisternum body and/or manubrium (Figure 2) to reproduce anatomical reduction. Six to eight 2.7 mm self-tapping stainless steel screws secure the plates into position using a 1.5 mm drill bit. After that, eight-gauge stainless steel sternal wires are placed transversely beneath the reconstruction plates while the hemisternums are reduced with bone-approximating clamps. Finally, the wires are tightened, and the sternal defect is closed. 

**Figure 3:**
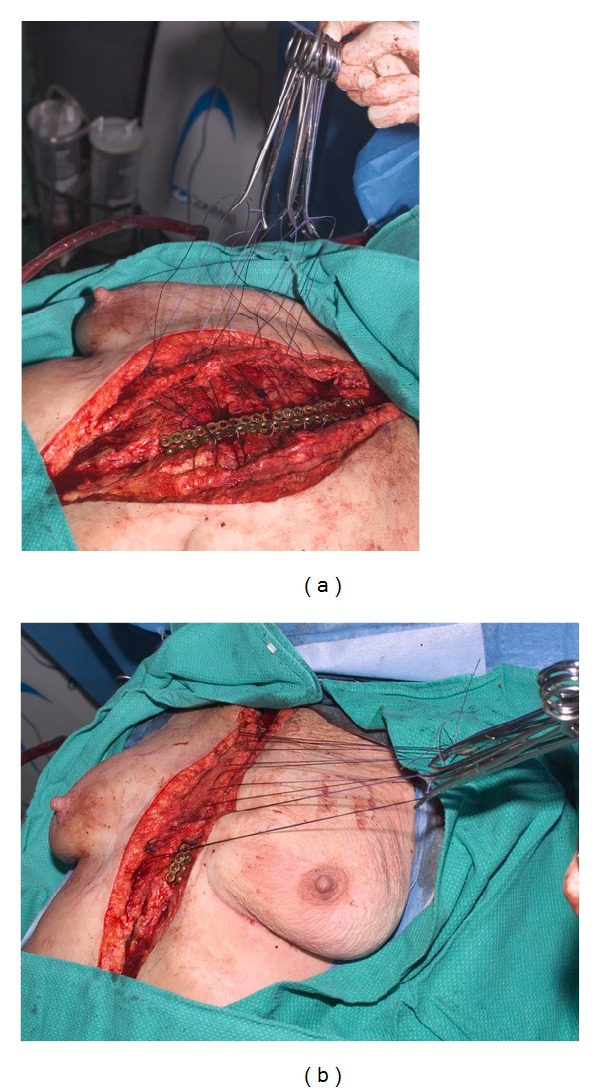
(a) Bilateral pectoralis major muscles are elevated from the anterior chest wall and subsequently (b) anatomically reapproximated to close the dead space overlying the reduced sternum.

After sternal reduction and fixation, bilateral pectoralis major muscle and overlying soft tissue are dissected from their insertion along the medial aspect of the ribs to the level of the midclavicular line until being mobile enough for the approximation in the midline (Figure 3). Cautery would divide the intercostals perforating vessels leaving the muscle flaps nourishment mainly by their thoracoacromial vessels. Then, the pectoralis major muscle flaps are approximated in the midline using 0 polydioxanone II (Ethicon, Inc., Somerville, NJ, USA) heavy absorbable suture over two closed-suction drains closing the dead space overlying the reduced sternum. Furthermore, in order to prevent the formation of a seroma the suture of the approximated pectoralis major musculocutaneous advancement flaps is looped beneath the sternal wires, thus fastening the flaps to the chest wall. 

**Figure 4:**
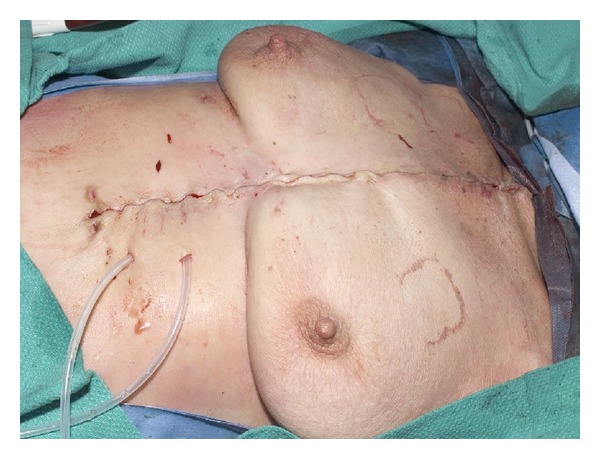
Two Jackson-Pratt no. 10 drains are placed one under each muscle flap. Skin closure is performed in three layers using Vicryl sutures.

After flap closure, two Jackson-Pratt no. 10 drains are placed, one under each muscle flap. Finally the deep fascia, subcutaneous tissue, and then skin are closed using interrupted absorbable sutures. Then, the drains are sutured to the skin and connected to bulb suction (Figure 4). After surgery, the patient is transferred to the ICU on sternal precautions and observation. Extubation occurs after surgery when the patient is otherwise stable. Drains are removed when the output is less than 20 cc for at least three days. Appropriate antibiotics therapy is ensued according to the organisms grown from the wound. As per the infectious disease consultant, a total of 6–8 weeks of intravenous antibiotics are administered in the case of osteomyelitis.

## 3. Methods

This study is in accordance to the declaration of Helsinki on the use of human subjects for research. Our institutional ethics review board also approved this study. Since August 1999 to the present, we have been using the technique of sternal rescue-open reduction and rigid internal fixation (ORIF) to correct postoperative mediastinitis following median sternotomy. A retrospective chart review was performed to compare the outcome of patients that had ORIF following postoperative sternotomy mediastinitis during a five-year period in our institution. These ORIF patients were compared with patients that did not undergo ORIF but only had sternal debridement and flap advancement surgery. Specific factors of preoperative medical and surgical treatments were noted along with any comorbidities, duration of hospitalization, days in the Intensive Care Unit (ICU), and days on a ventilator. The senior author performed all the reconstruction. The Student's *t*-test was used to compare means and a *P*-value of <0.05 was considered statistically significant. 

## 4. Results

All patients (*n* = 38) undergoing mediastinitis poststernotomy reconstructive operation from 1999 to 2004 were included in the study. All the patients were considered for the ORIF procedure. However, due to the excessive need of debridement leaving little or no sternum due to severe infection and necrosis, some patients (*n* = 29) did not undergo ORIF which we will name the control group. This has left close to a third of the patients (*n* = 9) undergoing the ORIF procedure, which we have named the experimental group. The indications for surgery were sternal dehiscence with infection (osteomyelitits and/or mediastinitis) in 70% of our patients and sternal dehiscence without infection in 30%. We found that 71% of the patients that underwent open reduction internal fixation had an infection associated with sternal dehiscence. In the control group, 69% of the patients had an infection associated with sternal dehiscence. 

The most common comorbid condition in the cohort of patients was coronary artery disease found in 98% (Table 1). The majority of the patients were male 58%, leaving 42% as female. The mean age was 64 years (range 44 to 82). The previous median sternotomy was performed for coronary artery bypass in 36 patients and for aortic valve replacement in 2 patients. Both internal mammary arteries were used in 9 patients, whereas only the left internal mammary artery was used in 18 patients and the right internal mammary artery in 1 patient. The most common organism grown was *Staphylococcus* species, and appropriate antibiotic therapy was ordered as per our infectious disease consultant (Table 2). All the patients were monitored two years postoperatively by the principle surgeon. 

It was found that only one of the ORIF patients was discharged from hospital before the diagnosis of mediastinitis was made. Preoperative wound drainage was found in 17% (*n* = 5) of the patients in the control group as compared to the 22% (*n* = 2) of the experimental group. Preoperative sternal instability and consequent breathing impairment were noted in 65% of the control group compared to the 87.5% (*n* = 8) of the ORIF experimental patients. Postreconstruction of the sternal instability decreased to a much greater degree in the experimental group than the control group (Table 3). Furthermore, it was noted that preoperative sternal motion pain in the control group decreased from 48% (*n* = 14) to 20% (*n* = 6) postoperatively. This is compared to the experimental group where there was a greater decrease in sternal pain postoperatively from 67% (*n* = 6) to 11% (*n* = 1) (Table 4). Postoperatively, the ORIF patients (experimental group) remained in the Intensive Care Unit (ICU) and on a ventilator for a mean of 1.5 and 0.75 days, respectively. Patients treated without ORIF (control group) spent significantly more days in the ICU (mean of 7.5 days, *P* < 0.05) and on a ventilator (mean of 2.15 days, *P* = 0.1). (Table 5).

Our postop complications included one hematoma in the ORIF patient group. There was one hematoma, one seroma, and four recurrent wound dehiscences in the control group (Table 6). There was one mortality in the traditionally treated group (control): an 85-year-old man who could not be weaned off the ventilator and subsequently died from respiratory failure. 

## 5. Discussion

 The goals for the management of sternal dehiscence include early diagnosis, wound closure, eradication of dead space and infection, and most importantly the protection of mediastinal structures. Consequently, the treatment which satisfies these goals and which is most popular amongst plastic and reconstructive surgeons is muscle-flap coverage and primary closure after wide sternal debridement [11, 12, 17]. However, this technique does not address a very important goal that is often neglected in the classic teaching. We believe the additional goal should be the restoration of sternal stability to maintain physiologic function and decrease pain-associated morbidity using open reduction and rigid internal fixation of the sternum. 

 The longitudinal placement of the stainless steel reconstructive plates over each hemisternum as opposed to the transverse placement of the plates across the sternal ribs is found to be superior in our center for the following reasons: (1) using the longitudinal placement, there is a reduction of the transverse fractures on each hemisternum from the original closure with the sternal wires by the cardiothoracic surgeons. This allows not only the anatomical reduction but also a physiologic one as the fractures are reduced and let to heal appropriately. (2) Using the transverse plate placement entails high potential complications such as pneumothorax, intercostals vessel and nerve injury, and injury to the internal mammary artery and veins accounted for by the close proximity of these vital structures and the plates overlying the ribs. 

 Despite rigid fixation and restoration of the physiologic components to the thorax, ORIF for the treatment of mediastinitis following median sternotomy does have its limitations. The wound bed should be eradicated of any gross infection, for example, before foreign body material such as the titanium plating can be placed to prevent seeding and recurrence of infection. In such cases, it may be prudent to treat the infection with multiple surgical wash outs and debridement, vacuum assisted closure, and secondary ORIF once the infection appears to have subsided [18–21]. Furthermore, a stable solid sternal bony base is vital for the plate fixation. In cases where the infection and sternal wires have eroded, the majority of the sternal bone ORIF may not be possible. Many authors have recently advocated primary sternal wiring as a paradigm shift for cardiac surgeons with promising results with decreased infection and need for reoperations [22–24]. Both primary and secondary platings of the sternum have been associated with decreased infection rates, hospitalizations, and need for reoperations [13, 25–28]. Furthermore, there is a documented increase in bony union with the use of sternal plating systems [25, 29, 30]. Moreover, proper patient selection is paramount for the success of ORIF as a treatment of mediastinitis following median sternotomy. 

 To our best knowledge, this study is the largest series of patients who underwent postmediastinitis ORIF in Canada. We conclude that this technique described to manage mediastinitis significantly decreases morbidity and mortality. It provides anatomic reduction as well as physiologic stabilization. Furthermore, there has been an elimination of infection, and it provided a technique for wound closure in a single stage operation. We have shown that ORIF will improve the quality of life of the patient by minimizing abnormal sternal mobility and pain and will also decrease inpatient costs by decreasing days spent in the ICU and ventilator dependence. Moreover, with proper patient selection, ORIF as a treatment of mediastinitis following sternotomy can improve patient outcomes and overall costs. 

## Figures and Tables

**Table 1:** The most common comorbid condition in the cohort of patients was coronary artery disease found in 97%. Combination of factors present in 29 patients.

Comorbid factors	Number of patients (%)
Coronary artery disease	37 (97%)
Hypertension	18 (60%)
Hypercholesterolemia	11 (37%)
Diabetes mellitus	10 (33%)
Obesity	5 (17%)
Chronic obstructive pulmonary disease	5 (17%)
Renal insufficiency	3 (10%)

**Table 2:** Different organisms were grown in the sternal wounds with the most common being of the *Staphylococcus species*.

Organisms	Number of patients (%)
*Staphylococcus* species	7 (47%)
Methicillin resistant *Staphylococcus aureus *	1 (7%)
*Clostridium *	2 (13%)
*Proteus *	2 (13%)
*E. coli *	2 (13%)
Fungi	1 (7%)

**Table 3:** Preoperative sternal instability decreases to a much greater degree in the ORIF experimental group than it does in the control group.

	Control	ORIF
Preoperative sternal instability	65%	87.5%
Postoperative sternal instability	25%	11.1%

**Table 4:** Preoperative sternal pain decreases to a much greater degree in the ORIF experimental group than it does in the control group.

	Control	ORIF
	Number of patients (%)	Number of patients (%)
Preoperative sternal pain	14 (48%)	6 (20%)
Postoperative sternal instability	6 (67%)	1 (11%)

**Table 5:** The experimental group (ORIF) has a significantly less time spent in ICU after reconstructive operation than does the control group. There is also a trend of being less ventilator dependent in the experimental group as compared to the control group.

	Control	ORIF	*P*-value
ICU (days)	7.5	1.5	<0.05
Ventilation (days)	2.15	0.75	<0.1

**Table 6:** There are more complications in the control group including 1 seroma, 1 hematoma, 4 recurrent infections, and one mortality as compared to only 1 hematoma in the ORIF experimental group.

Complication	Control	ORIF
	Number of patients (%)	Number of patients (%)
Hematoma	1 (3%)	1 (19%)
Seroma	1 (3%)	0 (0%)
Recurrent sternal wound dehiscence	4 (14)	0 (0)
Death	1 (3%)	0 (0%)
